# A comparison of patients receiving vertebral body tethering for adolescent idiopathic scoliosis in the public and private hospital setting

**DOI:** 10.1186/s13018-024-05254-1

**Published:** 2024-11-22

**Authors:** Samuel Ng, Zhang Changmeng, Jason Cheung, Graham Ka Hon Shea

**Affiliations:** Department of Orthopaedics and Traumatology, Li Ka Shing Faculty of Medicine, The University of Hong Kong, Pok Fu Lam, Hong Kong

**Keywords:** Vertebral body tethering, Scoliosis, Healthcare setting, Outcomes, Complications

## Abstract

**Purpose:**

Vertebral body tethering (VBT) is a new growth-modulating surgery for adolescent idiopathic scoliosis (AIS) requiring a distinct skillset and intraoperative setup. We compared perioperative details and outcomes of VBTs performed in a public pediatric orthopedic hospital and a general private hospital setting.

**Methods:**

We identified all patients receiving VBT for AIS from 1/2020 to 12/2023 with ≥ 6 months post-operative follow-up, with surgeries performed by the same senior surgeons. Clinical, radiological, and surgical details were retrieved.

**Results:**

24 VBTs were performed in the private setting and 16 in the public setting. Average age at operation was 11.9 ± 1.1 at a Sanders staging of 3.8 ± 1.2 when the major curve Cobb angle was 50.5 ± 8.0°. Tethered curves were most often thoracic in location (23/40) followed by thoracolumbar/lumbar curves (10/40) and double curve tethers (7/40). Overall correction ratio of 68.0 ± 19.0% was achieved. Time from booking to operation (82.2 ± 39.2 vs 63.1 ± 34.4 days, *p* = 0.112) and operation time (310 ± 86.4 min vs. 289 ± 87.4 min, *p* = 0.054) were longer in the public and private setting respectively but failed to reach statistical significance. Time to chest drain removal (1.5 ± 0.8 vs. 3.5 ± 1.7 days, *p* < 0.001) and length of stay (4.3 ± 0.9 vs. 6.6 ± 1.8 days, *p* < 0.001) were significantly shorter in the private setting, whilst complication rates remained similar (7/24 vs. 3/16, *p* = 0.456).

**Conclusion:**

Expertise, resource availability, and costs differ in the public and private healthcare setting. With regards to VBT, the conditions for referral and surgical outcomes remained similar. Earlier drain removal and discharge for patients managed in the private setting was not associated with an increase in complication rate.

## Introduction

Scoliosis refers to a coronal deformity of the spinal column of at least 10° (Cobb’s angle) on standing posteroanterior (PA) radiographic films. Despite this definition, it is well known that scoliosis is a three-dimensional deformity involving a rotatory element [1]. Adolescent idiopathic scoliosis (AIS) is the most prevalent cause of coronal deformity in the paediatric population with a prevalence of 1–4% [2]. Untreated AIS may continue to progress into adulthood and result in worsening deformity, chronic pain, cardiopulmonary compromise, and impaired psychological health [3]. For curves larger than 50°, there is a consensus to offer surgery to correct the deformity since that natural history regarding curves of this magnitude is to continue to worsen [4].

Surgical treatment of AIS by means of internal fixation and spinal fusion has made remarkable progress over past decades, in large part due to an improvement in instrumentation as well as techniques for deformity correction. Although spinal fusion can correct the underlying deformity and prevents curve deterioration, its sequalae include reduced spinal mobility and accelerated degeneration over remaining motion segments. Hence, growth modulating and non-fusion techniques such as vertebral body tethering (VBT) have been introduced. The principle of VBT, in addition to achieving immediate correction via tension applied to the convex side of the spinal curvature, is to slow remaining physeal growth via the Hueter-Volkman principle [5]. Indications for VBT continue to evolve but include thoracic or thoracolumbar/lumbar curves in skeletally immature patients, and prompt intervention prior to a Sanders staging of 4–5 is essential to achieve growth modulation [6].

Clinical professoriate staff at our academic institution perform VBTs in both the public and private hospital setting. The former comprises of a specialized orthopaedic hospital with a team of experienced anaesthetists, scrub nurses, operation theatre assistants, and radiographers with considerable spine surgery experience to facilitate the complex operative workflow. However, the government subsidized public setting is overburdened with potential delay in arranging theatre time. In contrast the private sector has ready availability of theatre and ward resources yet is offset by lack of expertise in spinal surgery workflow and financial considerations. We hypothesized that an overburdened public setting would have delayed scheduling of VBT surgery in a timely manner, whilst conversely the infrastructure in the private setting which was lacking in experienced supporting staff would have compromised the technical capability of the surgeon to accomplish similar outcomes and as short a surgical time as those managed in the public setting. Whilst many previous studies have analysed surgical correction and post-operative complications associated with VBTs, the objective of this study was to compare referral conditions, inpatient stay, and peri-operative outcomes of procedures according to setting of care.

## Materials and methods

### Study cohort and inclusion criteria

This was a retrospective study on prospectively collected data and approved by the local institutional review board of both a public teaching hospital as well as a private hospital affiliate. University Professoriate possessed practice rights at both institutions, where they attended to patients in the clinic, ward, and operating theatre. Consent was waived as anonymized patient data was utilized. We identified patients receiving VBT for AIS from January 2020 to December 2023. This starting date for recruitment conformed to when VBTs were performed with regularity by our unit, whilst the end date allowed patients a minimum of 6-months of follow-up after surgery. As all patients operated upon within this period were recruited, this precluded  pre-hoc sample size calculation. All procedures were performed by the same two senior surgeons. We excluded cases receiving hybrid surgery (VBT and posterior spinal fusion). At our centre, the surgical indication for VBT was AIS curvatures ranging from 40° to 70°, with a skeletal maturity ranging from Sanders grade 3 to 5.

### Intraoperative details

Patients underwent surgery using implants from either REFLECT™ (Globus Medical) or The Tether™ (ZimVie). A minimally invasive thoracoscopic-assisted approach was favoured, with screw and tether placement via two separate incision per curve. Exposure to the thoracic spine was facilitated by single-lung ventilation, and to the thoracolumbar/lumbar spine via the use of bronchial blockers. Chest drains were placed for all thoracic curves and thoracolumbar curves. Patients managed in both settings were managed by the same enhanced recovery after surgery (ERAS) anaesthetic protocol. Total intravenous anaesthesia was provided via propofol, whilst ketamine and panadol were given intra-operatively as preventive analgesia. The post-operative pain regimen consisted of patient-controlled anaesthesia at up to post-operative day (POD) 1, as well as regular oral Panadol, celebrex, oxynorm, and lyrica. Patients were encouraged to feed on POD 0. Urinary catheter removal was performed on POD 1, at the time when patients received mobilization and walking exercises under supervision by the physical therapist.

### Clinical data retrieval

From electronic operation and anaesthetic records, we retrieved patient weight (kg), operation time (minutes), intra-operative blood loss (mls), curves tethered (single thoracic / single thoracolumbar or lumbar / double curve tether), and levels instrumented. From ward and nursing notes, we retrieved details on the day of chest drain removal, drain output (mls), length of hospital stay (days), and Scoliosis Research Society (SRS)−22 scores. The timing and details of complications were retrieved following review of all inpatient and outpatient records after surgery.

### Radiological assessment

Pre-operative left-hand X-rays and whole spine standing X-rays were analysed for skeletal age (Sanders staging) [12] and Cobb angles respectively [11]. Post-correction Cobb angles were assessed on post-operative standing X-rays when patients were independently ambulatory and fit for discharge. The correction ratio was calculated by the formula (major curve pre op Cobb angle – post op angle) / major curve pre op angle. If the VBT procedure involved tethering of two curves, the correction ratios of both curves were determined and analysed as separate entities.

### Statistical analyses

Statistical analysis was performed using SPSS (IBM, version 26.0) and R (R Core Team, version 4.3.0). Continuous variables were expressed as mean values ± standard deviation, with intergroup comparisons performed using Student’s t-test. Categorical variables were expressed as the number of affected subjects, in which Pearson’s Chi-square and Fischer’s Exact tests were used for comparison. Multivariate correlation analyses were utilized for outcomes with continuous variables, while logistic regression analyses were applied to categorical outcome variables. All missing data were excluded from analysis. For all tests conducted, the level of statistical significance for two-tailed testing was set at p ≤ 0.05.

## Results

### Patient demographics

A total of 40 patients were included in the study, consisting of 24 VBTs performed in the public sector, and 16 in the private sector. The mean age at operation was 11.9 ± 1.1 years which did not vary in accordance with site of care (*p* = 0.559). There was a female predominance amongst patients from both private and public hospitals (20/24 vs 15/16, *p* = 0.329). The mean weight at operation was 39.2 ± 7.4 kg which did not differ between groups (*p* = 0.246). The mean pre-op Sanders maturity score was 3.8 ± 1.2 which also did not differ between groups (*p* = 0.372). Twenty-three (57.5%) patients had VBT on a single thoracic curve, while 10 patients (25%) had VBT on a single thoracolumbar or lumbar curve. The remaining 7 (17.5%) VBT performed on two curves. The mean number of vertebral levels tethered per curve was 6.9 ± 1.3, which did not differ by site of care (*p* = 0.303). Regarding pre-operative radiological parameters, the mean Cobb angle was 50.5 ± 8.0° which did not differ between groups (*p* = 0.793). However, subgroup analysis demonstrated that thoracolumbar / lumbar curves were larger amongst patients operated upon in the private sector (52.6 ± 7.4° vs. 47.9 ± 4.4°, *p* = 0.029) which contributed towards larger correction ratios (79.4 ± 15.1° vs. 66.9 ± 16.6°, *p* = 0.018). Details are summarized in Table 1.

**Table 1:** Demographic, clinical, and radiological details

	Overall	Private hospital	Public hospital	*P*-value
Patient number	40	24	16	
Gender				0.329
Male	5	4	1	
Female	35	20	15	
Age at OT (yr)	11.9 ± 1.1	12.0 ± 1.1	11.8 ± 1.1	0.559
Time from booking to OT (days)	70.9 ± 37.1	63.1 ± 34.4	82.2 ± 39.2	0.112
Pre-op weight (kg)	39.2 ± 7.4	40.3 ± 8.5	37.5 ± 5.1	0.246
Pre-op Cobb’s angle (o)	50.5 ± 8.0	50.2 ± 6.7	50.9 ± 10.1	0.793
Thoracic curves	50.5 ± 8.8	49.2 ± 6.3	53.6 ± 13.0	0.161
Thoracolumbar/lumbar curves	50.4 ± 6.5	52.6 ± 7.4	47.9 ± 4.4	0.029
Pre-op Sanders staging	3.8 ± 1.2	3.9 ± 1.2	3.6 ± 1.2	0.372
Curves tethered				0.051
Thoracic curve	23	15	8	
Thoracolumbar / lumbar curve	10	3	7	
Both curves tethered	7	6	1	
No. of vertebral levels per tether	6.9 ± 1.3	7.0 ± 1.1	6.6 ± 1.50	0.303
OT time (min)	289 ± 87.4	310 ± 86.4	256 ± 80.8	0.054
Thoracic curve	275 ± 57.4	277 ± 51.3	273 ± 71.3	0.878
Thoracolumbar / lumbar curve	237 ± 77.6	278 ± 70.0	220 ± 78.7	0.305
Double VBT	405 ± 87.2	411 ± 94.2	373	–
Blood loss (mL)	175 ± 153	155 ± 114	203 ± 193	0.329
Thoracic curve	179 ± 141	160 ± 106	215 ± 194	0.385
Thoracolumbar / lumbar curve	149 ± 190	83.3 ± 104	177 ± 218	0.508
Double VBT	220 ± 147	200 ± 173	280	–
Time with chest drain (days)	2.3 ± 1.6	1.5 ± 0.8	3.5 ± 1.7	< 0.001
Chest drain output per day (mL)	178 ± 74.9	196 ± 73.7	150 ± 70.2	0.056
Length of hospital stay (days)	5.2 ± 1.7	4.3 ± 0.9	6.6 ± 1.8	< 0.001
SRS-22 scores				
Pre-op	88.43 ± 6.87	85.57 ± 5.22	91.29 ± 7.48	0.123
6 months post-op	94.65 ± 12.29	94.56 ± 11.54	94.80 ± 14.05	0.963
12 months post-op	95.75 ± 9.68	97.56 ± 7.25	92.12 ± 13.15	0.201
Post-op Cobb’s angle (o)	16.2 ± 10.0	14.3 ± 9.05	19.6 ± 11.0	0.104
Thoracic curves	17.8 ± 10.4	15.6 ± 8.94	22.8 ± 12.3	0.038
Thoracolumbar / lumbar curves	13.5 ± 9.01	11.3 ± 9.10	16.0 ± 8.83	0.114
Correction ratio (%)	68.0 ± 19.0	71.6 ± 17.5	61.5 ± 20.2	0.101
Thoracic curves	64.8 ± 19.7	68.3 ± 17.7	56.7 ± 22.8	0.078
Thoracolumbar/lumbar curves	73.5 ± 16.6	79.4 ± 15.1	66.9 ± 16.6	0.018
Complications	10/40	7/24	3/16	0.456
Over/under-correction requiring surgery	3/40	1/24	2/16	0.553
Tether breakage	4/40	3/24	1/16	0.638
Other	3/40	3/24	0/16	–

### Comparison of peri-operative and post-operative outcomes for VBTs performed in private and public hospital setting

The mean operation time was 289 ± 87.4 min. Overall operation time was longer in the private hospital (310 ± 86.4 vs. 256 ± 80.8 min, *p* = 0.054). We considered patient weight, number/location of curves tethered, and the number of vertebrae per tether towards multiple regression of operation time with accordance to hospital setting. This similarly failed to show a significant effect of hospital setting upon OT time (*p* = 0.618). Mean intra-operative blood loss was 175 ± 153 mL, which did not differ between treatment setting (*p* = 0.329). Day of chest drain removal was significantly earlier in the private than public sector (1.5 ± 0.8 vs. 3.5 ± 1.7 days, *p* < 0.001). The mean length of hospital stay was shorter in the private compared to the public sector (4.3 ± 0.9 vs. 6.6 ± 1.8 days, *p* < 0.001). Length of hospital stay remained reduced in the public setting after multivariate regression accounting for OT time, number/location of curves tethered, and number of vertebrae per tether (*p* < 0.01). Post-operative Cobb angles amounted to a mean of 16.2° ± 10.0° which did not differ between groups (*p* = 0.104). A similar correction ratio was achieved amongst groups, amounting to 68.0 ± 19.0% compared to pre-operative Cobb angles (*p* = 0.101). There were no significant differences for SRS-22 scores pre-op (*p* = 0.123), at 6 months (*p* = 0.963), and 12 months post-op (*p* = 0.201).

A total of 10 (25%) patients had complications at the time of latest follow-up. Tether breakage was most common (4/40, 10%), followed by under correction / overcorrection (3/40, 7.5%). Revision surgery was required in 4/40 patients (10%) at the time of latest follow-up. Complication rates were similar in the private and public setting (7/24 vs. 3/16, *p* = 0.456). After adjusting for patient weight, curves tethered, vertebral levels per tether, OT time, and length of hospital stay in multivariate analysis, complication rates remained statistically insignificant with regards to treatment setting (*p* = 0.127). Details of each complication are listed in Table 2.

**Table 2:** Summary of complications

Public/Private	Age of OT	Gender	Curved tethered	Levels	OT time (min)	Pre-op Cobb’s	Post-op Cobb’s	Complication	Time of complication (months after OT)	Readmission
Private	13	F	1	T11-L4	300	57	21	Tether breakage	25	N
Private	11	F	1	T9-L3	200	49	− 6	Overcorrection	13	Y (cut tether)
Private	15	M	1	T9-L3	360	60	17	Tether breakage	3	N
Public	11	F	1	T8-L2	275	52	16	Under correction	30	Y (PSF)
Public	10	F	1	T10-L3	367	52	34	Tether breakage	23	Y (PSF)
Public	11	F	1	T6-L1	231	52	13	Under correction	24	Y (PSF)
Private	11	F	2	T5-11, T11-L3	420	48, 52	23, 11	Tether breakage	17	N
Private	12	F	2	T5-11, T11-L3	390	45, 42	22, 2	SMA syndrome requiring parental feeding	0.5	Y
Private	11	F	2	T5-11, T11-L3	435	43, 55	12, 3	Pleural effusion	0.5	N
Private	11	M	2	T6-11, T11-L4	285	35, 57	8, 7	Lower limb temperature asymmetry due to sympathetic chain disruption	0	N

**Fig. 1:**
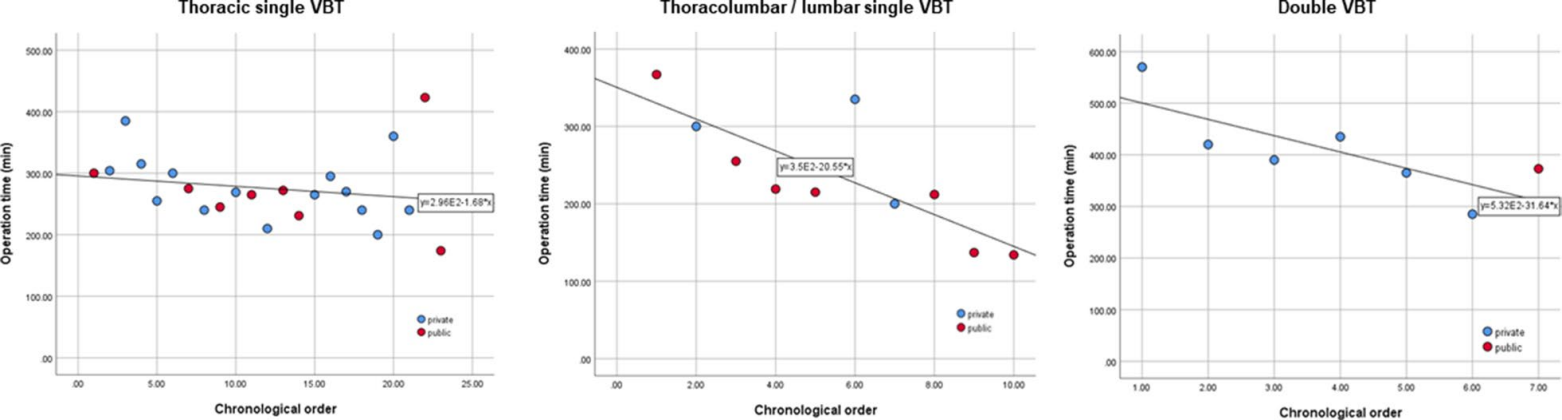
Scatterplots of operation time with accordance to chronological order of surgical date

There was a trend towards decreased operation time when the chronological order of the procedure was considered regardless of hospital setting (Fig. 1). Gradient of the best fit line and corresponding R^2^ value indicated that the reduction in surgical line time was most apparent for double curves (− 31.6 min / procedure, R^2^ = 0.615), followed by thoracolumbar / lumbar VBTs (−20.6 min, R^2^ = 0.642) followed by and thoracic VBTs (− 1.68 min, R^2^ = 0.039). No discernible trend in operation time in procedures performed in public and private hospitals was observed.

### Comparison of outcomes for single vs. double VBTs

As summarized in Table 3, patients receiving single and double VBTs did not differ by gender (*p* = 0.182), age at operation (*p* = 0.819), blood loss (*p* = 0.439), time with chest drain (*p* = 0.952), length of hospital stay (*p* = 0.556), pre-op Sanders staging (*p* = 0.340), or correction ratio (*p* = 0.637). Unsurprisingly, those receiving double curve VBT required a significantly longer operation time (405 ± 87.2 vs. 264 ± 65.4 min, *p* < 0.001). A higher complication rate was also noted following double curve VBT (4/7 vs. 6/33, *p* = 0.031).

**Table 3:** Single vs double VBT

	Single curve	Double curve	*P*-value
Patient number	33	7	
Gender			0.182
Male	3	2	
Female	28	5	
Age at OT (yr)	11.9 ± 1.1	11.8 ± 0.8	0.819
Time from booking to OT (days)	75.3 ± 39.0	51.9 ± 20.4	0.134
Pre-op weight (kg)	39.4 ± 7.8	38.4 ± 5.7	0.750
No. of levels	7.1 ± 1.3	6.9 ± 2.3	0.753
OT time (min)	264 ± 65.4	405 ± 87.2	< 0.001
Blood loss (mL)	170 ± 155	220 ± 147	0.439
Time with chest drain (days)	2.3 ± 1.6	2.3 ± 1.8	0.952
Drain output per day (mL)	172 ± 74.7	203 ± 76.3	0.327
Length of hospital stay (days)	5.2 ± 1.6	5.6 ± 2.1	0.556
Complications	6/33	4/7	0.031
Over/under-correction requiring surgery	3/33	0/7	–
Tether breakage	3/33	1/7	0.552
Others	0/33	3/7	–
Pre-op Sanders staging	3.7 ± 1.2	4.2 ± 1.0	0.340
Correction ratio (%)	67.1 ± 19.3	70.0 ± 18.7	0.637
Thoracic curves	67.4 ± 20.9	56.5 ± 13.6	0.080
Thoracolumbar/lumbar curves	66.5 ± 16.2	83.5 ± 12.0	0.001

## Discussion

Vertebral body tethering (VBT) is a recent advancement in the surgical management of scoliosis demonstrating the capacity for immediate deformity correction with further improvement following remaining skeletal growth. Prior studies have emphasized peri-operative complications [7] and mid-term efficacy [8] towards achieving safe and consistent deformity correction whilst expanding upon procedural indications. The emphasis of this study was to compare peri-operative and post-operative outcomes in VBTs performed in the public and private sector. The medical infrastructure at our locale comprises of a heavily subsidized and overburdened public sector whereby citizens pay $USD 15 per day as an inpatient regardless of treatment received, whereas expenses for scoliosis surgery in the private sector often exceed $USD 50 000 and are not completely recompensed by insurance policies. Clinical professoriate employed by the university hospitals are the only doctors having practicing rights in both the public and private healthcare sectors. To the best of our knowledge, this is the first study investigating the effect of healthcare setting on VBT referral and outcomes.

Intra-operative setup for VBT greatly differs from traditional posterior spinal fusion in that patients are positioned laterally, the lung may have to be partially collapsed, minimally invasive retractors, and an endoscope is required. Frequent X-ray evaluation of staple and screw position is mandatory. Patients receiving tethering for both curves require re-positioning of both the patient as well as equipment. For these reasons we hypothesized that the workflow would be expedited when performed with support from experienced personnel at the specialized orthopaedic public hospital. We identified a steady decrease in operating time for double VBTs and thoracolumbar / lumbar curves with accumulated caseload, but no discernible difference with regards to hospital setting. These results imply that the limiting factor to both expedited as well as safe surgery was the senior surgeon’s learning curve. The decrease in operation time for our present study was more drastic than a related study reporting improvement in 30 min after every 10 cases owing to the surgical learning curve [9]. Another study demonstrated that average surgical time fell from 390 to 163 min between the first 20 and last 20 cases of a 90 patient cohort [10] and similarly from 4.8 to 3.3 h upon CT guidance [11], with decrease demonstrable even after VBTs had been performed for 5 years. Our surgical time was comparatively longer likely because of a lesser case load, with 3 h being a reasonable target upon accumulated experience for single curve tethering.

Earlier drain removal in the private sector was the result of surgeon preference as well as better access to radiographic evaluation. Within the public setting, on-site and emergent radiological evaluation were unavailable after office hours and on weekends, and the initial working protocol for residents performing daily ward rounds was to remove the chest drain only when output was lower than 100 ml per day. Within the private sector, the senior surgeon performed daily rounds and preferred early chest drain removal at a less stringent output threshold of < 200 ml per day. There was no obvious trend towards an increased incidence of pulmonary complications with earlier drain removal. The single case with significant pleural effusion upon follow-up was performed in the private sector, with drain removal on day 1 after an accumulated output of 120 mls. We have now standardized our post-operative protocol for removal of the chest drain on post-op day 1 if daily drain output was less than 200 mls. The initial drainage output arises from residual saline irrigation within the wound cavity, leading to an overestimation of ongoing output. A recent study has described the use of smaller bulb suction drains instead of chest drains for patient comfort and reduced patient length of stay [12].

Earlier discharge from the private sector was likely the result of expedited drain removal, financial burden of prolonging stay, as well as availability of physiotherapists for rehabilitation. Post-operative physiotherapy was not routinely available over the weekend in the public sector, slowing mobilization and confidence for discharge. The length of stay following VBT has been reported by others to range from 3.6 to 8 days [13, 14], with the former study showing that stay duration was 1.4 days less than that of a matched case receiving PSF [13]. Our cohort, with an average a length of stay of 4.3 ± 0.9 days for those treated in the private sector, adds to this body of literature for supporting a comparable early discharge time [15, 16]. Despite a significant burden on the public healthcare system resulting in long overall elective operation waiting times [17], it was reassuring that VBTs in both public and private healthcare setting had a similar waiting time from booking date to surgery, as ensuring a period of sufficient remaining skeletal growth is essential towards successful growth modulation. Satisfaction was also comparable based on SRS-22 scores.

Waiting time and socioeconomic aspects have been considered for AIS within existing literature. The impact of delayed surgery on AIS patients scheduled to receive posterior spinal fusion is well documented, with one study describing an average waiting period of 24 months resulting in curve progression of over 25 degrees, an increase in surgical time by two hours, and an extra day of stay as an inpatient [18]. Scheduling of VBT must be expedited to ensure the patient is still skeletally immature, with time from consultation to surgery reported to be 74.8 days in an American study, comparable with our cohort, albeit with a range from 22 to 246 days [19]. This resulted in conversion in surgical plan from VBT to fusion in 4/95 patients, as most studies have selected patients with Sanders staging of 5 or below as a tethering threshold [20]. A cost effectiveness study identified VBTs to be more expensive than posterior spinal fusion to perform, but VBTs resulted in higher quality-adjusted life years [21].

Limitations to this study included a lack of details on mid- to long-term outcomes regarding correction until skeletal maturity and associated complication / revision rate since our follow-up duration of a minimum of 6 months only accounted for short term outcomes. Study size was limited by the operative load within the recruitment period. Power analysis exceeded 0.9 for time with chest drain and of hospital stay but was only 0.48 and 0.67 for time from booking to OT and correction ratio respectively. With larger patient numbers, the assessment of non-inferiority, for example with regards to minimal clinically important differences for SRS-22 scores, would also have been informative [22]. The impact of out-of-pocket financial expenses upon early discharge were not explored, but an important question to address in locales with substantial disparity between public and private healthcare resources.

In conclusion, this study was the first to compare VBTs performed in public and private hospitals by the same senior surgeons. Length of stay and chest drain insertion was longer in the public sector. Otherwise, operative arrangements, intraoperative execution, and outcomes were similar regardless of setting.

## Data Availability

No datasets were generated or analysed during the current study.
